# *Drosophila* as a Robust Model System for Assessing Autophagy: A Review

**DOI:** 10.3390/toxics11080682

**Published:** 2023-08-08

**Authors:** Esref Demir, Sam Kacew

**Affiliations:** 1 Massachusetts General Hospital, Harvard Medical School, Boston, MA 02129, USA; 2 F.M. Kirby Neurobiology Center, Boston Children’s Hospital, 300 Longwood Avenue, Boston, MA 02115, USA; 3 Department of Neurobiology, Harvard Medical School, Boston, MA 02115, USA; 4 Medical Laboratory Techniques Program, Department of Medical Services and Techniques, Vocational School of Health Services, Antalya Bilim University, 07190 Antalya, Turkey; 5 R. Samuel McLaughllin Center for Population Health Risk Assessment, Institute of Population Health, University of Ottawa, 1 Stewart (320), Ottawa, ON K1N 6N5, Canada; sam.kacew@uottawa.ca

**Keywords:** *Drosophila melanogaster*, autophagy, in vivo animal model system, Atg, fat body, development

## Abstract

Autophagy is the process through which a body breaks down and recycles its own cellular components, primarily inside lysosomes. It is a cellular response to starvation and stress, which plays decisive roles in various biological processes such as senescence, apoptosis, carcinoma, and immune response. Autophagy, which was first discovered as a survival mechanism during starvation in yeast, is now known to serve a wide range of functions in more advanced organisms. It plays a vital role in how cells respond to stress, starvation, and infection. While research on yeast has led to the identification of many key components of the autophagy process, more research into autophagy in more complex systems is still warranted. This review article focuses on the use of the fruit fly *Drosophila melanogaster* as a robust testing model in further research on autophagy. *Drosophila* provides an ideal environment for exploring autophagy in a living organism during its development. Additionally, *Drosophila* is a well-suited compact tool for genetic analysis in that it serves as an intermediate between yeast and mammals because evolution conserved the molecular machinery required for autophagy in this species. Experimental tractability of host–pathogen interactions in *Drosophila* also affords great convenience in modeling human diseases on analogous structures and tissues.

## 1. Introduction

### 1.1. The Morphology of Autophagy

**Figure 1:**
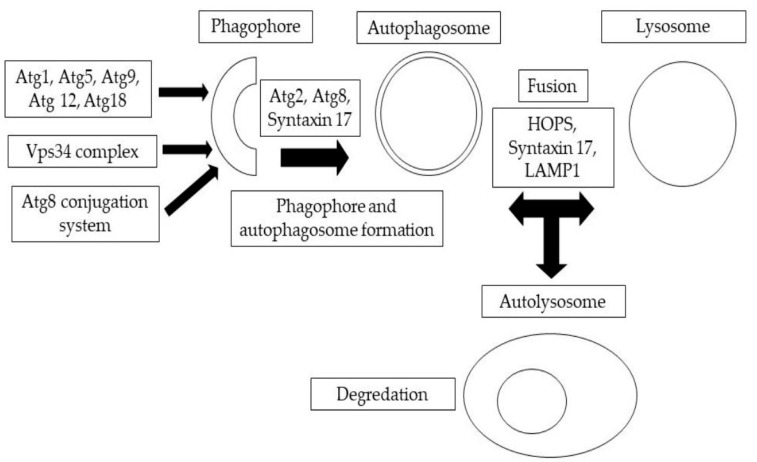
A summary of autophagic structures. The development of phagophores and double-membrane autophagosomes is mediated by the sequential and coordinated activity of Atg protein complexes.

Eukaryotic cells naturally destroy and recycle damaged cellular components through a process known as autophagy, which is activated by a variety of environmental and developmental triggers [1]. In eukaryotic cells, the main protein degradation pathways are the proteasome and lysosomal breakdown. Autophagy is a lysosomal degradation process that can be classified into different routes based on how the intracellular material reaches the lysosome. Mammalian cells exhibit three different versions of autophagy, namely, microautophagy, macroautophagy, and chaperone-mediated autophagy, each distinguished by their morphological characteristics [2,3]. During macroautophagy, large portions of cytoplasm are engulfed by a membrane cistern called a phagophore or isolation membrane, which then forms a double-membrane autophagosome [3]. The anatomical steps of autophagosome formation are illustrated in Figure 1. This autophagosome then fuses with the endolysosomal components for degradation. In microautophagy, late endosomes (lysosomes) directly engulf tiny portions of the cytoplasm. Chaperone-mediated autophagy is another process that allows individual proteins to reach the lysosomal lumen by means of Hsc70 and the lysosome-associated membrane protein 2A (Lamp-2A) in mammalian cells [3,4]. It is worth noting that, as there is no homolog for the protein Lamp2A in *Drosophila melanogaster*, chaperone-mediated autophagy induced by Lamp-2A will not take place. In all forms of autophagy, the cargo material is degraded by acidic lysosomal hydrolases, and the resulting monomers are recycled back to the cytosol for use in energy production and biosynthesis [4]. Autophagy serves a critical function in maintaining cellular homeostasis by responding to stresses or disturbances such as nutrient starvation. When the biogenesis of autophagosomes is inhibited, it leads to the accumulation of selective autophagic cargo, including Ref(2)P/p62-positive aggregates of polyubiquitinated proteins. These aggregates can be cytotoxic and can contribute to cellular dysfunction [5]. It has been observed that autophagy is often impaired in cancer cells, leading to uncontrolled cell growth [4]. Similarly, autophagy dysfunction, or misregulation, has been linked to several neurodegenerative disorders [4,5]. This review focuses specifically on macroautophagy, which is a bulk degradation pathway conserved over the course of eukaryotic evolution and responsible for the clearance of whole organelles, long-lived cytosolic proteins, and aggregates within eukaryotic cells [6,7]. Neurodegenerative illnesses are becoming more prevalent in older populations, which has a devastating impact on both individuals and their communities. Khurana and Lindquist [8] showed that *Saccharomyces cerevisiae*, a developing yeast, plays a special role in the arsenal against neurodegeneration. *S. cerevisiae*, as a basic eukaryotic organism, providesinteraction-scale mechanistic insights into cell-autonomous neurodegenerative pathways. To recognize and describe these components, a number of PD models, including non-mammalian eukaryotic creatures, have been established. Surguchov [9] has discussed recent discoveries in three model organisms for Parkinson’s disease, including yeast, *Drosophila*, and the nematode *Caenorhabditis elegans*, which revealed unique processes and pinpointed fresh causes of the condition. These non-mammalian models and human cells function similarly in many conserved molecular and cellular pathways.

### 1.2. Homologous Autophagy Proteins between Yeast/Drosophila/Mammals

The proteins responsible for the formation of autophagosomes were first discovered in the yeast *S. cerevisiae* and are referred to as Atg (Autophagy-related) proteins [10]. These proteins have counterparts in higher eukaryotic organisms, such as *D. melanogaster*, and their roles are highly similar [11]. The identification of the Atg proteins marked a significant step forward in understanding autophagy. Specifically, 18 Atg proteins in *Drosophila* make up five complexes that operate the autophagic process in this species [12,13]. Investigations in yeast have uncovered 33 genes, referred to as ATG, that play a role in autophagy. Much of this genetic material has been preserved in organisms with increased complexity [11,14]. The formation and expansion of the autophagosomal membrane, which encloses cellular components for degradation, is regulated by the ATG proteins that are conserved across eukaryotic organisms [15,16]. The fusion of autophagosomes with lysosomes, which occurs through the action of Rab7 and the binding of HOPS and SNARE proteins in yeast, is a crucial step in the autophagic process [17]. It is noteworthy that the processes of autophagosome–lysosome fusion and the subsequent degradation of autophagic cargo vary significantly between yeast and animal cells. However, the HOPS tethering complex and Rab7 are conserved across *Drosophila* and mammals [18,19,20]. Additionally, in animal cells, autophagosome degradation also requires the Rab2 protein [12,21], unlike in yeast where SNAREs are not homologous [22,23]. Furthermore, molecular motors such as dynein and kinesins play a role in the movement of autophagosomes to lysosomes for degradation [24,25].

The term “autophagy” describes a set of procedures used by eukaryotic cells to recycle and degrade cellular components. The Atg1 complex regulates autophagy’s molecular mechanism, which has three main stages: initiation, the production of autophagosomes, and expansion and completion of the membrane [26]. Autophagy is activated by the Atg1 complex in all eukaryotic organisms. The formation of autophagosomal membranes is led by the Vps34 complex, and the autophagosome membrane expands depending on two distinct ubiquitin-like protein conjugation machineries involving Atg8 and Atg12–Atg5. After autophagosome completion, vesicles merge with lysosomes, creating autolysosomes. In this respect, Table 1 highlights the central autophagy genes from humans and baker’s yeast (*S. cerevisiae*), and their counterparts in *D. melanogaster* [27]. Each model has its own advantages or limitations. *Drosophila* is an excellent genetic model but a poor one for biochemistry and physiology, while mammals present difficulties for large-scale genetic screening. Yeasts are single-celled and do not go through development.

The Atg1 protein, a serine/threonine kinase found in various organisms, has a crucial role in the process of autophagy. Studies have shown that Atg1 is necessary for autophagy in mammalian cells [28,29,30,31] and *Drosophila* [29,89], nevertheless the composition and function of the Atg1 complex varies between species [41]. For instance, in yeast, the TOR pathway regulates the formation of the Atg1-Atg13-Atg17 [41] and controls autophagy induction through phosphorylation of Atg13. When nutrient levels are low, the dephosphorylation of Atg13 increases its affinity for Atg1-Atg17, triggering autophagy [41]. Neither Atg17 nor Atg29 and Atg31, which both interact with Atg17, are present in equivalent form in either *Drosophila* or humans [43,78]. In contrast to yeast, the Atg1 and Atg13 proteins in *Drosophila* and mammals constitute a more stable structure independent of the activity of the TOR pathway. The Atg1 ortholog in mammals also exhibits interactions with Atg13 independently of the nutrition or starvation status [34,46]. Furthermore, *Drosophila* orthologs of both Atg101 and FIP200, necessary for autophagosome generation in mammals, have been identified but not yet tested for their role in autophagy [34,45,46]. Not only do the *Drosophila* Atg1 and mammalian unc-51-like kinase 1 (ULK1) complexes have different functions but also the overexpression of *Drosophila* Atg1 triggers autophagy, whereas the overexpression of Ulk1 in mammals suppresses it [32,38]. The source of the disparity is uncertain; theories have been proposed that attribute it to the effect of extra regulatory proteins [90,91]. In contrast, Atg101 is found in the majority of eukaryotes apart from budding yeast. It has been suggested that the control of autophagy initiation by the Atg1 complex and its regulatory mode may have developed from yeast to animal cells [90]. Specifically, previous work indicates that Atg101 mutants have reduced lifespan, increased oxidative stress, and impaired mitochondrial function, and Atg101 is required for autophagy, a process that is known to be involved in age-related processes [92]. These findings suggest that Atg101 may be a key regulator of tissue homeostasis and aging in *Drosophila*.

After the Atg1 complex triggers autophagy in yeast, *Drosophila*, and mammalian cells, a PI3P-enriched structure may be seen where autophagosomes develop. PI3P is a phospholipid manufactured by the enzyme PI3K (phosphatidylinositol 3-kinase), whose role in the formation of autophagosomes (membrane-bound vesicles used to transport cellular components for degradation) is still unknown. However, it is thought that PI3P may recruit additional components to the autophagosome, as several yeast proteins that bind to PI3P and localize to the autophagosome have been shown to depend on PI3K activity [93]. Although there are several proteins involved in autophagy, only one of them, namely Atg18, has an ortholog that is found in both *Drosophila* and mammals. Additionally, it has only been confirmed that the ortholog found in *Drosophila* is essential for autophagy to occur [32,79].

The yeast PI3K complex, a team of vital proteins including Vps34, Vps15, Atg6, and Atg14 [94], are the masterminds behind the formation of autophagosomes. These proteins are not only present in yeast but also in *Drosophila* and mammals [95]. Like *Drosophila*, mammals too have three versions of PI3K, but the type III PI3K, Vps34, stands out by activating autophagy through its production of PIP3 [96]. Likewise, Vps34, a member of the class III PI3K family, has a role in initiating autophagy through the production of PIP3. However, things get a bit more complicated in mammals, as the Vps34-Vps15-Atg6 team can be found working alongside other proteins like Ambra1, Atg14, Rubicon, and UVRAG [95]. Even though the *Drosophila* body contains proteins comparable in function to UVRAG, Rubicon, and Atg14, which are found in the Vps34 complex in other organisms, it is not currently understood how these proteins function in the Vps34 complex in *Drosophila*. They may play a similar role as in other organisms or they may have unique functions, and further testing is needed for more evidence on their role in the Vps34 complex in *Drosophila*.

Vesicle expansion is a process that allows the vesicles (small, enclosed spaces within a cell) to grow in size, mediated by two groups of proteins known as ubiquitin-like groups: Atg5-Atg12-Atg16 and Atg8. These groups have remained largely unchanged throughout the evolution from yeast to mammalian organisms [97]. The Atg16 complex is a collection of proteins that localizes to the autophagosome and plays a crucial role in the formation of the autophagosome membrane. The autophagosome is a structure that encloses cellular components that are intended for destruction [84,98]. The Atg12 protein is covalently conjugated to Atg5 through a process that resembles the conjugation of ubiquitin to a target protein, which involves two other proteins called Atg10 and Atg7. The second of these corresponds to a protein called E1 enzyme that is responsible for activating ubiquitin involved in the ubiquitin conjugation process [72]. Atg10 is a protein contributing to the generation of the Atg5-Atg12 complex, which functions similarly to an E2 ubiquitin-conjugating enzyme, a protein that plays a role in the ubiquitin system, but it is not related to those found in the ubiquitin system [99]. Once the Atg5-Atg12 complex is formed, it is linked to Atg16 in a non-covalent manner to form the completed complex [84]. Nevertheless, despite the presence of orthologs in *Drosophila* for each of these proteins, further experiments are warranted to show whether the orthologs of Atg10 and Atg16 play a part in the autophagy pathway in *Drosophila*.

The other conjugation structure involved in autophagosome generation is a process that links Atg8 to a type of lipid called phosphatidylethanolamine (PE) through an amide bond [66]. This process helps in the formation of the autophagosome membrane, which is essential for the autophagosome to enclose the cellular materials that are targeted for degradation. The Atg8–PE conjugation is crucial in the formation and expansion of the autophagosome. Conjugation of Atg8 to PE begins with the action of the cysteine protease Atg4, which cleaves Atg8 [69]. Once Atg8 is cleaved, it is bound to Atg7. In the next phase, Atg8 is transported to Atg3, which is an enzyme comparable to E2 ubiquitin-conjugating enzymes, which then catalyzes the conjugation of Atg8 to PE. Humans have four proteins that are equivalent to yeast ATG8: ATG8L, GATE16, MAP1LC3, and GABARAP. These human orthologs of yeast ATG8 are also conjugated to PE in a comparable fashion to the way it occurs in yeast [67,81]. In *Drosophila,* there are two genes that code for Atg8 proteins (Atg8a and Atg8b) that are found in the autophagosomes [29,82,83]. Such proteins probably have some overlap in function, as deleting the Atg8a gene results in a less severe phenotype than what would be expected for a protein that has such a vital part in autophagosome formation [32,79]. *Drosophila* also has equivalents of Atg3, Atg4, and Atg7, which have been proven to play a part in the tightly regulated pathway of autophagy [29,68,100].

### 1.3. Drosophila as a Model

Comparative genetic research revealed a significant degree of genetic similarity between humans and a variety of other animals, including *D. melanogaster*. It has been estimated that roughly 60% of the fly genome is comparable to that of humans and that approximately 77% of known disease genes in *Homo sapiens*, including those involved in diabetes, autism, and carcinoma, have matching sequences in *D. melanogaster* [101], which highlights the value of this species in exploring human biology and relevant risk factors [102,103].

This means that the *Drosophila* fruit fly can be used in a variety of experiments because it is adaptable and can be used in combination with other testing methods, which renders it an ideal choice with optimal adaptability and versatility for larger-scale experiments, such as RNAi or mutant screens, requiring a large number of samples [104]. Genome sequencing has greatly facilitated identifying *Drosophila* genes that are almost identical to human genetic material associated with certain diseases. Previous cDNA (complementary DNA) analyses [105] detected 289 of these corresponding genes in fruit flies, and around three-quarters of the genes causing certain human diseases, such as diabetes, autism, and cancer, were found to have a functional corresponding gene in *Drosophila* [102,103]. In this regard, FlyBase, a database containing data on the genetics, genomics, and biology of *D. melanogaster*, has more than 800 reports that detail the links between specific human diseases and *Drosophila* genes, offering researchers valuable insight into the genetic basis of human diseases, which may lead to the development of new treatments and therapies (FB2018_03) [106]. Another important advantage of *Drosophila* studies is that they are exempt from the ethical limitations associated with the use of more complex organisms such as mammals [107]. In that vein, the principles of humane experimental technique known as the 3Rs (replacement, refinement, and reduction), which have been widely accepted as guidelines for humane animal treatment in toxicology experiments, can be readily put into practice with this species [108]. The fundamentals of cell biology, from gene expression and neuron synapse formation to cell signaling and differentiation, are shared between humans and *Drosophila* flies. Even immune signaling pathways, when exposed to cytokines, remain remarkably similar between the two species [109]. The GAL4/UAS system, in combination with CRISPR, has been effectively modeled by employing *Drosophila* as a dynamic tool [110,111]. CRISPR’s tissue-specific genome editing can confine mutations to specific cells or tissue [110], making research on flies applicable to vertebrates, including humans. More recently, Trinca and Malik [112] have investigated the impact of gamma radiation on autophagy using *Drosophila* as a testing model, discovering that exposure to radiation resulted in elevated autophagy levels in two different cell types (gut and brain), which suggests that autophagy might have a function in the initial response to exposure to radiation.

While *Drosophila* offers a range of benefits as an experimental model organism for research on human biology, there are also several limitations to consider. One such limitation is that the difference in typical body temperatures of adult fruit flies and humans (18–27 °C versus 36–37 °C) may result in multiple variations in host–pathogen interactions. Additionally, the lack of certain factors in *Drosophila* can limit the types of pathogenesis studies that can be conducted using this organism. Furthermore, the lack of sialic acid as a main surface molecule on fly cells may prevent the study of certain viruses that depend on this molecule for entry into host cells [113]. Another key limitation to consider is that *Drosophila* has a less complex immune system compared to mammals. At the same time, in comparison to mammals, flies are much cheaper to maintain, can be mailed by standard post, and do not require authorization to transport. Thus, despite such drawbacks and restrictions, *Drosophila* remains a valuable model organism for researchers in gaining insights into basic biological processes before moving on to more complex testing models [114,115,116].

### 1.4. The Life Cycle of Drosophila 

**Figure 2:**
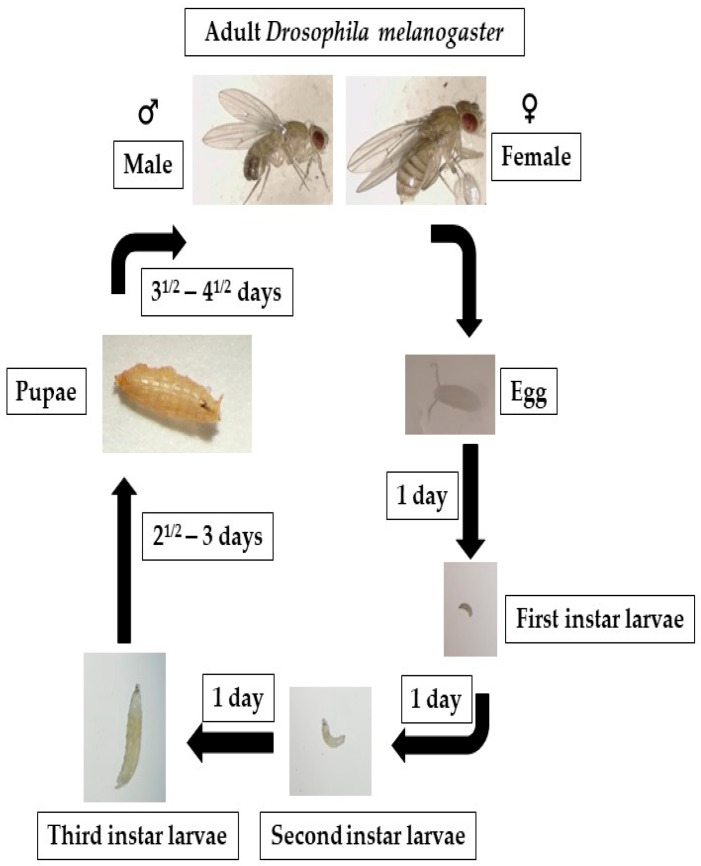
Fruit flies go through four distinct life stages: embryo, larva, pupa, and adult. After 6 to 8 h, the eggs begin to hatch, producing first-instar larvae that develop into second- and third-instar larvae. The larvae turn into pupae around day five. On the ninth or tenth day, the fruit fly enters its adult form.

*Drosophila,* a holometabolous insect, follows a developmental process characterized by prepupal and pupal stages of immobility during which the entire organism undergoes a complete metamorphosis. *Drosophila* exhibits a rapid life cycle (9–10 days at 25 °C) (Figure 2). This process entails the histolysis of larval tissues, and diploid cells undergo proliferation and differentiation to generate adult organs, which are fully developed by the time of emergence as an imago from the pupal case. In the context of fruit fly development, three distinct larval stages, L1, L2, and L3, can be identified and are separated by highly regulated molting transitions [117]. At the mid-L3 stage, a slight elevation in levels of 20-hydroxyecdysone in combination with levels of juvenile hormone causes an alteration in larval behavior, wherein they exit their food source and commence searching for an environment to metamorphose, and developmental autophagy triggered by ecdysone occurs in the majority of tissues in the larvae that have multiple sets of chromosomes [83]. In autophagy, the cells in such tissues break down and recycle their own components for freeing up stored biological matter, which can be used as an energy source by diploid cells in the case of programmed cell death. Autophagy helps ensure that the cells have enough energy and nutrients to complete this metamorphosis process [83,118]. It is likely that autophagy plays a significant role during metamorphosis, a period of developmentally regulated starvation that spans five days. Also occurring during the remodeling of muscles in pupae, autophagy is thought to contribute to the DNA fragmentation in cells that provide nutrition stability to their neighboring cells (nurse cells) during egg cell formation [21,119].

### 1.5. Research Tools Available for Drosophila

In *Drosophila*, the visualization of autophagy involves detecting the presence of Atg8, a protein that is found in autophagosomes in mammals, flies, and yeast, which suggests that autophagy is a conserved process across species and that Atg8 is a valuable marker for detecting autophagy in different organisms [73]. *Drosophila* Atg8 antibodies instrumental in the distribution, localization, and expression levels of proteins in cells and tissues are not readily available, but the Gal4/UAS system, a powerful tool for gene expression in *D. melanogaster*, allows for tissue-specific or inducible expression of target genes by using a Gal4 transcriptional activator. By using a GFP-tagged version of Atg8, researchers can study the distribution, localization, and expression levels of Atg8 in specific tissues or under specific conditions. The GFP tag makes it possible to visualize Atg8 in living cells, which helps researchers understand the dynamics of autophagy processes [29,47,83]. While GFP-tagged Atg8 transgenes have been widely used as indicators of autophagosome formation, we should remember that there are some concerns over the validity of this approach. One concern is that the GFP-tagging of Atg8 may interfere with its normal function in autophagy, potentially leading to inaccurate conclusions about autophagosome formation. Additionally, some studies have reported that GFP-tagging can alter the localization and distribution of Atg8 within cells, further complicating the interpretation of results. Therefore, it is important to consider these potential limitations when interpreting results obtained using GFP-tagged Atg8 reagents and to corroborate findings with other methods [120]. In order to overcome the limitations associated with Atg8-GFP-based autophagosome detection, many *Drosophila* researchers supplement it with additional assays, such as lysosomal markers (e.g., LysoTracker Red, LAMP1). By using multiple markers, researchers can get a more comprehensive and reliable understanding of autophagosome formation and autophagy activity. In addition to using multiple markers, another correlative measurement of autophagic activity involves measuring transcriptional upregulation of Atg genes [82,121,122]. These complementary approaches provide a more robust assessment of autophagy activity and help overcome the limitations associated with using Atg8-GFP as the sole indicator of autophagosome formation.

Electron microscopy (EM), despite being non-quantitative, is considered the gold standard for demonstrating the presence of autophagosomes, as it provides direct and high-resolution visualization of cellular structures; thus, it is still widely employed in autophagy research involving experiments on *Drosophila* tissues. Detecting autophagosomes in cells provides insight into their autophagy activity, but the interpretation of the results can sometimes be challenging. For instance, increased autophagosome numbers may point to a rise in autophagy or a failure of autophagosome–lysosome fusion that causes a decrease in autophagy functionality. This is particularly crucial when determining the connection between autophagy and disease development. Therefore, efforts are underway to evaluate autophagic flux, which monitors autophagosome–lysosomal degradation by tracking the movement of cytoplasm, organelles, and other cargo. For instance, researchers evaluated autophagic flux through Western blotting to analyze the time-course of ubiquitinated aggregates on samples from *Drosophila* brain [123]. They discovered that, as autophagic activity declines in the brain as it ages, while the levels of ubiquitinated proteins elevate and Atg8 gene mutations exacerbate the accumulation of ubiquitinated proteins, expression of Atg8 prevents aggregate accumulation. These findings demonstrate that ubiquitinated protein levels can be considered a reliable indicator of alterations in autophagic flux, providing valuable insight into the dynamics of autophagy in diseases and aging.

Transmission electron microscopy (TEM) may afford some valuable data on organelle ultrastructure but requires additional tests to analyze autophagy and flux, and may not be practical for high-throughput genetic screens involving analysis of large numbers of genes or genetic variations in a rapid and systematic manner, including RNA interference (RNAi) screens, CRISPR-Cas9 screens, and whole-genome sequencing studies. In this regard, confocal laser scanning microscopy (CLSM) stands out as a commonly employed alternative for analyzing autophagy in *Drosophila*, as it allows for relatively quick and efficient analysis of large numbers of cells or tissues, although it may not provide the same level of detail as TEM. Staining with vital dyes such as acridine orange, ethidium bromide, and propidium iodide are commonly adopted to detect the presence of autophagosomes and lysosomes, and can also be used to quantify the amount of autophagic flux [124]. Fluorescent reporters, such as GFP-LC3, can label autophagic vesicles and track the dynamics of autophagy in real time. Moreover, the reagents employed in research on autophagy in *Drosophila* samples are often identical or comparable to those used in higher vertebrates, facilitating comparison between different species [1].

A key factor in autophagy, Atg8a covalently conjugates to phosphatidylethanolamine in autophagosomes, which is considered a marker of autophagy. Atg8a is found in various organisms, such as fruit flies, yeast (Atg8), and mammals (LC3). Transgenic *Drosophila* lines with Atg8a expression tagged with GFP or mCherry can now be obtained from public stock centers, and such lines allow in vivo experiments on autophagy under both starvation and developmental conditions [29,83].

In addition to being a marker for autophagy, Atg8a also plays an essential role in autophagosome formation, during which cells create a membrane-bound vesicle to enclose cellular materials for degradation and the protein Atg8a covalently conjugates to phosphatidylethanolamine, a type of lipid. This binding is essential for the formation of the autophagosome and allows the enclosed materials to be degraded and recycled. An E1-like ubiquitin-activating enzyme, Atg7 activates ubiquitin-like protein conjugation systems, while Atg3, an E2-like enzyme, catalyzes the transfer of the ubiquitin-like protein from Atg7 to the substrate. The Atg12-Atg5-Atg16 complex is an E3-like enzyme that facilitates the transfer of the ubiquitin-like protein from Atg3 to the substrate. Atg8a’s advantage over other Atg proteins appears to be a greater amount of lipidated Atg8a remaining tagged along with completed autophagosomes, which suggests that Atg8a may have a role in the maturation of autophagosomes and may be important for their stability [15]. Atg8a forms a membrane anchor for autophagosome formation and maturation, where it is attached to the outer and inner membranes. In later stages of autophagosome development, Atg4 separates Atg8 from the external membrane, while it still stays bound to the inner membrane. Two abnormalities were consistently seen in V-ATPase-depleted fat body cells: a substantial increase in vesicle size in response to 4 h of starvation and an inappropriate buildup of mCh-Atg8a-labelled vesicles in the cells of well-fed animals [125].

By tagging LAMPs (lysosome-associated membrane proteins participating in the transfer of molecules into and out of the lysosome) with a fluorescent marker, lysosomes can be visualized in living cells and tracked over time, which allows researchers to study the dynamics of lysosomal trafficking and the role of lysosomes in autophagy and endosomal–lysosomal degradation regardless of their acidification status [12,20,126]. The mCherry-Atg8a reporter is particularly effective in labeling autolysosomes because it is targeted to the autophagosome and the fluorescent signal remains stable when it is transported to the lysosome. This reporter is therefore widely employed to specify autophagic structures, such as autolysosomes and autophagosomes, and in cases where autophagy is impaired, autophagosome formation is also impaired, resulting in no punctate signal being detected [127]. This fluorescence reporter can also indicate changes in the expression of autophagy-related proteins, which can be indicative of defects in autophagosome fusion or maturation [128]. The presence of large and bright autolysosomes in cells signals that they are undergoing normal cellular degradation processes, while small, faint autophagosomes in starved mutant cells suggest an inability to degrade contents efficiently [12,18,22,127].

Recent studies have reported switching to the use of *Drosophila* Atg8a fused to triple mCherry and LAMP reporters and that such triple-mCherry-tagged fluorescent reporters facilitate visualization of autophagic vesicles and lysosomes in fat body experiments and other tissues from larval wing disc or midgut cells of adult *Drosophila* [12,129].

The selective receptor of ubiquitinated proteins, commonly known as Ref(2)P/p62, is a remarkable autophagic cargo that can be tracked through fluorescent or HA-tagged reporters [20,39,130]. Not only can Ref(2)P/p62 be detected but it can also be the focus of autophagic degradation [5,131]. This means that when autophagy is functioning correctly, p62 levels are low, as it is quickly degraded; however, if autophagy is impaired, p62 accumulates and forms aggregates, and such accumulation can be visualized through the CLSM, allowing the measurement of autophagic degradation in a cell [12,20,39,132].

Lysotracker and Magic Red dyes are commonly used to stain fat cells in larvae [12,20,29,47,133], and are membrane permeable, accumulating in acidic organelles. The appearance of Lysotracker-positive vesicles in starved cells in cases of autophagy induced by starvation indicates a dramatic increase in the compartment known as an autolysosome [29,47]. Commercially available Magic Red dye, which fluoresces red upon intracellular Cathepsin B protease activity, can reliably identify and characterize functional lysosomes/autolysosomes that contain active cathepsin [20,133]. However, it is important to note that macrophages and nephrocytes also carry large endolysosomes or phagolysosomes, components in the elimination of cellular waste, including foreign particles and bacteria, through phagocytosis, which test positive for Lysotracker, so they may be confused with autolysosomes [134]. Hence, these dyes cannot be solely relied upon to indicate autophagy in such cells.

The lack of antibodies that allow the detection of specific endogenous proteins through Western blotting (WB) and indirect immunofluorescence (IF) methods, as well as examination of interactions between proteins by immunoprecipitation assays, is a drawback of *Drosophila* as a model organism. However, the widespread availability of antibodies against Atg8a, the most common marker, makes it feasible to perform WB and IF microscopy experiments [12,71,77,135,136,137]. In addition, commercially available anti-GABARAP antibodies in humans are another alternative for research involving WB and IF techniques in *Drosophila* [127,138]. Monitoring and detecting endogenous Atg8a-positive vesicles is a reliable method for identifying autophagosomes. There is also an antibody against the protein SNARE Syntaxin17 (Syx17), which is necessary for autophagosome–lysosome fusion. However, despite its localization in autophagosomes [23], anti-Syx17 antibody alone is not a definitive autophagosome marker, since it is also present in other organelles like mitochondria and endoplasmic reticulum [22,127]. Some commercially available anti-Atg5 and anti-Atg12 antibodies for *Drosophila* have been employed in studies using the IF method to track autophagy initiation as phagophore markers [12,23,139,140].

Handling fly tissues requires no special equipment, so IF methods could be performed in a fashion comparable to those employed while working on higher vertebrates. Fat bodies can be stained by inverting carcasses, fixing, and staining in small containers and then dissected and mounted after staining [127]. There are two different anti-Ref(2)P/p62 antibodies that may yield reliable results in studies employing WB and IF techniques [12,131,132].

The involvement of other intracellular vesicular transport pathways in autophagy may also be studied through markers for endosomes, the Golgi apparatus, and the endoplasmic reticulum. To that end, a comprehensive antibody toolkit was developed in Sean Munro’s lab [141]. In addition, fluorescently tagged reporters for organelles in transgenic *Drosophila* offer some potential to analyze autophagy progression and mutant phenotypes. When retromer depletion was tested on cells, lysosomal hydrolases were found to be loaded improperly, and enlarged acidic autolysosomes accumulated, which might have been mistaken for increased autophagy, although TEM studies revealed that the cytoplasmic material in these vesicles was still intact [142].

TEM is widely considered a standard method for characterizing vesicular transport events [12]. Although TEM is the gold standard for visualizing autophagic vesicles, it is imperative to validate its results using other methods such as the CLSM and biochemical assays [143], which provides additional information about autophagy and its impact on cellular processes. Autophagic structures in *Drosophila* cells are almost identical in appearance to those observed in mammalian cells, which makes it relatively straightforward for researchers with prior TEM experience to identify autophagic vesicles in *Drosophila*. 

Immuno-electron microscopy (Immuno-EM), as described by Lőrincz et al. [127], is another approach to the study of autophagy that can be performed through standard methods. Researchers often use acrylic resins, such as LR White, instead of epoxy resins, as well as milder chemical fixation methods to preserve the antigens. These adjustments are important to ensure that the antigens are preserved and can be successfully visualized and analyzed [23]. Also, utilizing an embedding method with a progressive temperature lowering may enhance the preservation of antigens during the Immuno-EM analysis [144]. As well as progressively lowering the temperature, techniques such as cryo-ultra-sectioning and sucrose infiltration of fixed samples may prove beneficial in immunogold labeling during such analyses. Another convenient tool could be correlative light and electron microscopy (CLEM), which combines the advantages of EM and light microscopy to provide high-resolution images of cellular structures and processes. Although CLEM demands task-specific equipment and expertise, it is appropriate for autophagy studies in *Drosophila* and can afford invaluable information about such complex biological processes. 

Another method is acid phosphatase enzyme cytochemistry, a technique widely adopted in the past to discover and analyze lysosomes in various organisms. It involves detecting the activity of acid phosphatases, which are commonly found in lysosomes and autolysosomes, through specialized histochemical staining procedures; thus offering a powerful way of visualizing the ultrastructural characteristics of lysosomes and autolysosomes, though it is now a largely forgotten technique. The practical method of acid phosphatase enzyme cytochemistry in *Drosophila* involves incubating fixed tissue samples with a substrate solution to visualize the presence and distribution of acid phosphatase in lysosomes within cells. The deposition of an electron-dense precipitate serves as a visual indicator of the lysosomal localization of the enzyme [12,127,144].

Western Blotting (WB) is typically conducted with samples obtained from the entire animal body or tissues dissected from the body. To begin, the samples are boiled in a Laemmli buffer containing SDS for three minutes, then homogenized. This boiling process is repeated to extract protein, and two centrifugation steps remove fat and other debris. Atg8a is the primary protein commonly detected in WB experiments [127]. It is comparable to the detection of mammalian LC3 in blots [1]. The method distinguishes between the autophagosome-associated version of Atg8a (Atg8a-II) and the non-lipidated form (Atg8a-I) by their migration during gel electrophoresis. Effective separation of the two bands using a 13% or higher polyacrylamide gel is used to evaluate autophagy. Increased levels of Atg8a-II protein as compared to a loading control suggest increased autophagosome numbers [12,18,20,23]. A decrease in the amount of Atg8a-II protein suggests a problem in the lipidation of Atg8a or induction of autophagy. At the same time, WB results require careful interpretation as high levels of lipidated Atg8a can accumulate in some Atg mutants and elevated levels could imply high autophagic activity in the cells [128]. To avoid misinterpretation, Atg8a immunoblots should always be evaluated along with flux and morphological assays.

WB research may also involve the detection of TOR (target of rapamycin) activation, which is known to inhibit autophagy. In such cases, researchers can determine phosphorylation levels of common TOR targets like 4EBP1 and S6K to estimate TOR kinase activity through readily available antibody kits like phospho-4E-BP1 and anti-phospho-S6K [12,125,138,145].

With uses comparable to mammalian RFP-GFP-LC3B reporters [1], tandem-tagged mCherry-GFP-Atg8a reporters are commonly employed to track autophagic flux in flies [12,23,119,146]. When autophagosomes fuse with lysosomes, the low lysosomal pH quickly reduces the GFP signal, thus autophagosomes are observed in the form of small dots that are positive for GFP and mCherry, while autolysosomes test positive for mCherry only. If internal material fails to degrade, enlarged yellow structures are observed under microscopy [18,23,128]. However, when a significant number of small autophagosomes are grouped together, it may complicate the visualization of each vesicle through the CLSM. In these instances, ultrastructural analysis can be beneficial in determining whether there is a fusion defect between autophagosomes and lysosomes or whether autolysosomal degradation is impaired. A common method for measuring autophagic flux involves examining the buildup of Ref(2)P/p62-positive protein aggregates and ubiquitinated proteins within cells [127,128,131,132]. Researchers have recently created a GFP-p62 reporter driven by the tubulin promoter to avoid issues with Gal4/UAS-mediated p62 overexpression and gene regulation. The reporter, expressed at a constant low level in larval tissues, primarily reflects autophagic degradation, making it a highly sensitive indicator of disrupted autophagic flux [12,20].

Ref(2)P/p62 antibodies are employed by researchers utilizing the WB method to monitor autophagic degradation where a rise in p62 levels often signifies a block in the autophagic intracellular degradation system [12,131,132]. Other techniques involving WB as the main tool for measuring autophagic flux exist, primarily relying on the transformation of tagged Atg8a reporters to free GFP or mCherry within lysosomes [127,128,147].

*Drosophila* has been used for medium-throughput drug experiments on a range of diseases such as heart dysfunction, neurodegeneration, and obesity induced by high-fat intake. *Drosophila* larvae and adult flies can be cultured in compounds like rapamycin to trigger autophagy in this model organism [12,29]. Spermidine, an autophagy-triggering compound, has been shown to increase lifespan in several models and protect against toxicity from pesticide paraquat, which has been linked to Parkinson’s disease (PD) and alpha-synuclein (α-Syn), a key protein found to play a role in the pathology of PD [127,148,149]. Toxicity assays have revealed that compounds mimicking ecdysone hormone in insects (like RH 5849) may initiate autophagy machinery in the fat body [12,83]. Chloroquine (CQ) is known to block the acidification of autolysosomes and trigger myopathies when fed to fly larvae, and this method of feeding CQ to larvae has been found to be effective in initiating muscle toxicity [12,150]. Bafilomycin A1 can also be utilized to prevent the merging and acidification of autophagosome and lysosome; however, due to the potential alteration of TOR signaling, caution must be exercised when evaluating the outcomes [12,151]. Additionally, further testing on flies could help validate possible drug candidates that may induce or enhance autophagy, such as AUTEN-67, known as autophagy enhancer-67 [12,152].

Another approach is to incubate fly tissues with drugs ex vivo, allowing for the determination of direct effects on a specific cell type and avoiding the potential toxicity of administering drugs to whole animals. Bafilomycin A1 is an example of a drug that can be tested using this strategy [125].

*Drosophila* is a valuable model system in part due to the abundance of genetic methods accessible to researchers without much effort. There are loss-of-function mutations available for almost all core autophagy-related genes. However, for the *Drosophila* counterparts of Atg101 and FIP200, which are newly discovered parts of the Atg1-Atg13 complex, no mutant alleles currently exist. This means that researchers do not have the tools to manipulate the function of these genes in the same way they can with the other core autophagy genes. In that regard, a database for *Drosophila* genetics, FlyBase, contains all information related to the current autophagy alleles and transgenic constructs, serving as a comprehensive resource for researchers to access up-to-date information on currently available genetic tools for autophagy research on *Drosophila*.

In *Drosophila*, tissues that are highly impacted by autophagy are postmitotic, which makes it challenging to conduct clonal analysis. This complicates the process of assessing the tissue-specific functions of autophagy genes, as their function is often pleiotropic. To overcome this challenge, RNAi technology has proven crucial, as it enables the precise control of gene knockdown in terms of both space and time. In contrast, genome-wide RNAi screens in *Caenorhabditis elegans* are commonly performed through feeding methods, a widely used practice in the study of gene function [153]. In *Drosophila*, RNAi feeding cannot be implemented, so alternative methods have been developed to produce transgenes expressing snapback/hairpin constructs. One of the most popular methods involves the utilization of Gal4/UAS [154,155,156] to specifically express a double-stranded RNAi hairpin, which results in the post-transcriptional silencing of target genes. Due to achievements reached by transgenic RNAi, research efforts are geared towards creating a large collection of hairpin lines on a genome-wide scale [155]. A hairpin construct is a piece of genetic material that folds back on itself to form a loop, with the two ends of the DNA strand base-pairing to form a hairpin-like structure. In this respect, researchers created a system for the targeted integration of these constructs into specific locations in the genome, allowing for the study of gene function and regulation. This system allows for the precise insertion of the hairpin construct at a specific location in the genome, allowing for the study of its effects on gene expression and cellular behavior [154,157]. Researchers collaborated with the *Drosophila* RNAi Screening Center to create a “second generation library” and produced transgenic RNAi lines that target the majority of the core autophagy genes, which are accessible on FlyBase [158,159].

The exact potential of using *Drosophila* genes for research on autophagy has yet to be understood. One reason for this is that the tissues most commonly targeted in autophagy studies on *Drosophila* are postmitotic and polyploid, making standard mosaic analysis less effective. Additionally, it is difficult to design a screening method for autophagy that does not have a readily observable morphology. While the RNAi-based evaluation of autophagy in well-known *Drosophila* cell lines shows some promise, there are questions about the biological relevance of these studies. The following section will discuss various approaches used for autophagy screening in *Drosophila*.

In the past, researchers demonstrated that changes in the *Drosophila* blue cheese gene (bchs) cause adult life expectancy to shorten as well as age-dependent brain degeneration and cell death [160]. Bchs and its human equivalent Alfy play a crucial role in the removal of protein aggregates through autophagy, although the exact mechanism is still unknown [161]. Studies employing stocks with deficiencies and chosen mutant variations managed to discover that mutations in the genes responsible for lysosomal trafficking altered the strong bchs eye appearance. Significantly, Atg1, Atg6, and Atg18 were found to accentuate the phenotype, implying that this method could accurately spot genes related to autophagy. Another study by Arsham and Neufeld [162] combined mosaic analysis and live-cell/fixed-cell imaging to screen autophagy regulators. They used the Flp-FRT system to generate homozygous mutant clones and analyzed the lysosomal activity in the mutant cells compared to surrounding wild-type tissue, identifying 79 transposon insertions heightening lysosomal activity. These studies highlight the importance of a multidisciplinary approach to the study of autophagy, as they demonstrate the importance of combining classical genetics with cutting-edge technologies such as microarray analysis and mass spectrometry. The results of these studies provide new avenues for further investigation into the mechanisms of autophagy in *Drosophila*. 

*D. melanogaster* is one of the best-studied models for numerous mutagenic screens, development, and aging. This fly proceeds through well-defined stages during its life cycle, including embryo, larva, pupa, and adult, accomplishing complete phenotypic metamorphosis. These changes are induced by carefully controlled gene expression at the transcriptional, epigenetic, and translational levels. Most developmental gene expression investigations using *Drosophila* are currently based upon RNA in situ hybridization and transcriptome analysis which employ large-scale microarray/RNA-seq data sets, or a combination ofboth. To that end, two contemporaneous studies explored genome-wide transcript analysis of salivary glands undergoing cell death related to autophagy [121,122]. By using microarrays, Lee et al. discovered several fly ATG genes that showed an increase in transcription, including Atg2, Atg4, Atg5, and Atg7. Further research from the same laboratory revealed that Dynein Light Chain 1 plays a crucial role in inducing autophagy during cell death in salivary glands [163]. The study by Gorski et al. [121] involved a serial analysis of gene expression (SAGE) approach to examine the transcript levels in salivary glands undergoing autophagic cell death. The authors reported that they identified over 732 differentially expressed genes with unknown functions. Juhasz et al. [68] used microarray technology to investigate the initiation of autophagy in the larval fat body and discovered that the downregulation of FK506-binding protein 39 kDa (FKBP39) occurred during autophagy. The study revealed that FKBP39 inhibited autophagy, probably by exerting a modifying or controlling effect on the Foxo transcription factor.

In addition to gene expression analysis, two research teams employed high-throughput screening by proteomic techniques to investigate proteins involved in autophagy in the salivary glands and fat body of *Drosophila*, allowing for comprehensive analyses of proteins expressed during autophagy, providing a deeper understanding of the molecular events underlying this cellular process [164,165]. The latter team employed a shotgun proteomics method to uncover the proteins involved in the autophagic cell death of larval salivary glands. Their findings aligned with earlier microarray and SAGE studies, but also revealed new players in the process—namely Warts, a kinase in the Hippo pathway that was found to be vital for the regulation of autophagy and the programmed cell fate of salivary glands [166]. The former team of researchers, Kohler et al. [164] utilized a mass spectrometry technique with an isotope-coded affinity tag to uncover the players in the starvation-prompted autophagic response. By contrasting proteins from starved and normal fat body in *Drosophila*, they managed to identify 110 proteins that varied in regulation. One noteworthy discovery was the upregulation of the lipid desaturase Desat1 in the starved sample, which was found to be a necessary component for starvation-triggered autophagy and to be localized to structures positive for Atg5 and Atg8.

*Drosophila* is a valuable testing model in research employing comprehensive genome-wide high-throughput RNAi screening thanks to its accessible cell culture lines that are capable of quickly absorbing long dsRNAs introduced to the medium, leading to effective target gene suppression [167]. Additionally, the availability of extensive dsRNA libraries enables large-scale screenings to systematically study the functions of all genes predicted from genomic sequencing. Despite this, there has been no published report of a genome-wide autophagy screening using this system, which is somewhat surprising. Nevertheless, it is evident that cell cultures from *Drosophila* can be utilized to explore autophagic function in immunity, cell fate, and nutrient deprivation [59,71,168,169]. In their screening study, the research team led by Chittaranjan aimed to validate the involvement of genes in autophagic cell death of the salivary gland. To achieve this, they selected 460 genes that had been previously identified through expression studies as potentially playing a role in this process. The screening effort was considered moderately sized, indicating a significant number of genes were being tested, but not necessarily covering the entire genome [170]. The researchers triggered cell death in a tumorous hemocyte cell line from *Drosophila* using ecdysone, known to initiate metamorphosis and autophagy-related cell death. Through dsRNA analyses, they discovered 25 genes that may affect survival. Further study showed that the knockdown of genes including Atg2, Atg3, Atg5, Atg6, Atg7, Atg8a, and Atg8b resulted in lower levels of cell survival [170]. The results of their study provided valuable insights into the genetic processes in salivary gland cells leading to death.

### 1.6. The Role of Autophagy in Organ System Function and Developmental Processes

Autophagy can also act in response to infection, and an autophagy protein called Beclin was demonstrated to provide some protection against Sindbis virus (SB)-induced encephalitis in mice [171]. Moreover, autophagosomes can directly engulf bacterial and viral pathogens [172,173,174]. Conversely, certain pathogens, including poliovirus and rhinoviruses, can use the autophagic machinery to replicate as well as escaping degradation by autophagy [175].

Although *Drosophila* lack the adaptive immune response present in higher vertebrates, this fly can still be a reliable, useful model organism for studying the function of autophagy in the immune system. In this regard, the Listeria infection highlights both benefits and drawbacks of bacteria-induced autophagy in adult *Drosophila*. Microorganisms responsible for listeriosis trigger antibacterial autophagy in fruit fly cells, relying on the peptidoglycan recognition protein LE [169]. However, Listeria can escape autophagy in mammalian macrophages through ActA in the cytoplasm and Listeriolysin O, creating non-degradable phagosomes [176,177,178]. Such phagosomes limit bacterial growth, forming from failed autophagosome or lysosome fusion attempts. Despite these varying autophagy responses to Listeria-induced infection, studying Listeria and autophagy in *Drosophila* cells can still provide insight into Listeria’s ability to evade autophagy.

Previous work in the field [71,179] once again confirmed the value of *Drosophila* as an experimental model in viral autophagy research. These studies found that vesicular stomatitis virus (VSV) infected both cultured and in vivo cells and triggered autophagy, and suppression of core autophagy genes with RNAi led to higher severity of VSV infection. Modifying the insulin signaling pathway in experiments was found to have an effect on virus replication, reflecting PI3K/Tor pathway’s contribution to autophagy. To date, this is the only study to examine virus-induced autophagy in fruit flies. Such a research model could attract further research due to *Drosophila*’s rapid genetics and the evolutionary distance between host and virus, making it easier to identify potential pathways involving interactions between pathogens and autophagy [180].

Autophagy is a vital process for cellular health, as it helps maintain a balance between the production and breakdown of cellular components as well as protecting cells from damage caused by various environmental factors. Autophagy also helps recycle nutrients and energy under starvation conditions, such as when oxygen levels are low, as is the case in the heart muscles of mammals, facilitating the survival of the cells [181]. Such a protective role could have considerable implications for tumor cells, because autophagy may either contribute to the survival of tumor cells [182] or inhibit their growth [183]. Conditions such as Parkinson’s, Alzheimer’s, and Huntington’s, which are all neurodegenerative diseases, involve the accumulation of large amounts of mutated proteins. Of all the human diseases related to autophagy, those concerning the unusual accumulation of proteins have the most advanced model systems in fruit flies, and increased autophagosome generation seen in such diseases could have protective functions [184]. Rapamycin is an immunosuppressant drug that has been shown to induce autophagy in some cell types. Rapamycin, an mTOR inhibitor, induces autophagy and speeds up the removal of these harmful substrates. Accordingly, treatment with the rapamycin analog has been found to cause declines in huntingtin aggregate accumulation and neurodegeneration. This suggests that induction of autophagy may be beneficial in the treatment of neurodegenerative diseases [185]. *Drosophila* neurodegenerative disease models have been employed in several studies to investigate the role autophagy plays in the toxicity of TDP-43, polyQ repeat, and Amyloid beta 42 [25,186,187,188]. By conducting a genetic analysis of the *Drosophila* version of Alfy, a protein associated with autophagy, researchers were able to verify its function in the context of Huntington’s disease. To further investigate how Alfy works, the researchers implemented Huntington’s pathological changes in the retina to examine its role in eliminating ubiquitin-positive protein inclusions and halting the degeneration of neurons. The results of this analysis showed that the *Drosophila* ortholog of Alfy is essential for such elimination and suppression of deterioration of neurons in vivo, confirming its role as a key player in autophagy. This research has been invaluable in furthering our understanding of the pivotal part of autophagy in the progression of Huntington’s and has provided insight into potential therapeutic strategies for this devastating neurological disorder [160,161,189].

### 1.7. How Data from Drosophila Informs Insights into the Role(s) of Autophagy in Mammalian Physiology and Pathogenesis

Autophagy is essential for keeping cells balanced and functioning normally, as well as helping them to cope with challenges like a lack of nutrients. An insect’s fat body stores fat and helps store and use nutrients and carries out vital metabolic activities, which means that it can be thought of as an insect’s equivalent of a liver [190]. The fat body, which serves as the primary storage location, acts rather quickly in cases of deprivation by releasing amino acids, carbohydrates, and lipids [190]. By exposing larvae to sucrose solution (20%) in the laboratory, autophagy in fat body cells can be triggered due to amino acid starvation. This method is advantageous in comparison to submerging larvae in water, as the solution’s high density enables larvae to float on the surface. Within an hour and a half, autophagy starts and it peaks in around three to five hours [29]. Larval exposure to sucrose solution has been found to trigger the synthesis of glycogen to great extents in fat cells, which is an interesting finding [191].

The fat body forms from the embryonic mesoderm and is made up of two lobes of cells arranged in monolayers for simple microscopic inspection. These benefits make fat cells a popular tool for researching autophagy in *Drosophila*. In addition, organs like the compound eye are also studied to explore the part of autophagy in neurodegenerative disorders [134]. In addition, the salivary glands are dependent upon both autophagy and apoptosis for death, while the midgut depends exclusively upon autophagy and represents the most reliable example of autophagic cell death in any organism. Autophagy in these tissues has been studied in both larvae and adults, allowing researchers to investigate the differences in autophagic processes between different developmental stages as well as the shrinkage and death of cells [79,192]. The larval midgut is an ideal organ for studying intracellular trafficking if epithelial cell polarization is desired, as it responds well to autophagy triggered by starvation in comparison to the fat body. Additionally, the ovaries of adult female *Drosophila* have also been used to analyze this type of autophagy [135].

Research into the Atg7-deleted mutant, the first autophagy gene null animal, showed a distinctive effect where *D. melanogaster* suffered delayed development, but no visible morphological defects. However, they were found to be more vulnerable to oxidative stress and nutrient deprivation, with a shorter life [82]. These phenotypes have been discovered in the presence of null mutants of certain genes like Atg5, Atg16, necessary for lipidation in *Drosophila* Atg8a protein, which is considered a homolog of mammalian LC3 proteins [136,193]. Lipidation in Atg8a and its mutants are both possible, likely due to the presence of residual autophagic degradation, which has been demonstrated in mammalian cells [194]. Overall, the function autophagy plays in aging and stress tolerance can be effectively studied using *D. melanogaster*. Research on a variety of model organisms, including mice, has shown that increased autophagy can help maintain cellular balance, thereby increasing longevity. For example, the expression of Atg8a and Atg1 in *Drosophila* neurons has been observed to extend lifespan by up to 50% in comparison to control animals. Additionally, moderate Atg1 expression in the fat body, intestine, and Malpighian tubules of *Drosophila* has been found to extend lifespan by altering mitochondrial genes and enhancing proteostasis [195]. The elevation in the amount of Ref(2)P (p62 in mammals), a significant cargo receptor in selective autophagy, not only enhances proteostasis but also mitochondrial function and mitophagy [196]. This mitophagy activation then leads to improved mitochondrial health and homeostasis, ultimately resulting in an extended survival or lifespan extension thanks to the activity of prolongevity pathways [197]. When mTOR is inhibited, a mitophagy-dependent decrease in cyclin E in germline stem cells (GSCs) and human induced pluripotent stem cells (hiPSCs) delays the normal G1/S transition, driving the cells toward reversible quiescence (G0) [33].

In *Drosophila*, while the selective autophagy of ubiquitinated proteins is understood well, organelle degradation has received little attention. However, Vincow et al. [198] used a proteomics-based method to show that the main autophagy pathway targets mitochondria for degradation in lysosomes, a cellular organelle acting as the cell’s recycling center. During this process, subunits of the respiratory chain, the series of protein complexes responsible for generating energy within the mitochondria, are selectively removed independently of the protein Atg7. The degradation of mitochondria in lysosomes can occur through the formation of mitochondria-derived vesicles. These vesicles are formed through a process that is dependent on the protein Syntaxin 17, although the precise details of this process have yet to be fully investigated in fruit flies. The formation of these vesicles and their subsequent fusion with lysosomes is thought to represent an important mechanism for the targeted degradation of mitochondria through autophagy [199,200].

Testing on complete animals instead of cultured cells in autophagy-related research offers a series of benefits; for instance, this approach allows us to observe the organism as a whole, such as through neuromuscular evaluations in negative geotaxis (climbing) assays, while it helps gain better insight into the intricate and tissue-specific regulation of autophagy by examining the information exchange between various tissues and cells through certain metabolites and hormones [129,138,201].

The ubiquitin–proteasome system and autophagy are two important mechanisms for maintaining protein quality control in cells. The ubiquitin–proteasome system acts like a “cleanup crew” for short-lived proteins, marking them for destruction with a chemical tag called ubiquitin and then breaking them down in a structure called the proteasome. Autophagy, conversely, can be thought of as a “recycling plant” for larger cellular structures and damaged organelles. Autophagy engulfs these structures in a membrane-bound sac, which then fuses with a degradation center called a lysosome to break down the contents and recycle the resulting molecules. In periods of starvation, autophagy serves as a homeostatic response to nutrient deprivation, whereas during periods of abundant nutrition, it is virtually undetectable in yeast; however, after about 30 min of nitrogen deprivation, autophagosome generation increases significantly. Thus, autophagy appears to emerge as a vital mechanism for maintaining cellular homeostasis in periods of nutrient deficiency [202]. The appearance of autophagosomes occurs within one hour of starvation in the fat body of the fruit fly (*D. melanogaster*), a multifunctional organ that performs functions similar to those of the liver in mammals in storing nutrients and providing a source of energy [29]. Similarly, research in mice has shown that under starvation conditions, autophagy increases in many organs as a way for cells to conserve energy and recycle cellular components for use as an energy source. This response is particularly pronounced in muscle tissue, where autophagy helps preserve muscle mass and prevent muscle wasting [203]. Additionally, it has been observed that autophagy is upregulated by a range of tissues in newborn mice, potentially as a means of adapting to the sudden lack of nutrients resulting from detachment from the placenta [84]. Thus, these findings demonstrate that the ability of autophagy to protect the organism in case of starvation appears to be a rudimentary function preserved during the evolution of eukaryotic organisms.

In yeast cells, the regulation of autophagy in response to starvation is primarily controlled by the Target of Rapamycin (TOR) pathway, which senses the availability of nutrients in the environment and adjusts cellular metabolism accordingly. Under conditions of high nutrient availability, the TOR pathway suppresses autophagy, allowing cells to grow and divide, whereas when nutrients become scarce, the TOR pathway is inhibited, leading to the activation of autophagy as a way for cells to conserve energy and recycle cellular components [96]. Similarly, higher eukaryotic organisms, such as *Drosophila*, also control and regulate autophagy by means of the PI3K pathway, which is upstream of the TOR pathway which senses changes in the levels of insulin and other growth factors and adjusts cellular metabolism [204]. Previous research confirmed that both the TOR and insulin pathways regulate autophagy in the fat body of *Drosophila* larvae [27]. The fat body cells of organisms have been observed to exhibit a rapid autophagic response when subjected to starvation, treatment with the drug rapamycin, or genetic inactivation of the TOR pathway [29,32,83]. This is evidenced by the strong induction of autophagy in this tissue following the loss of PI3K or insulin receptor function [29,32,83]. It has thus been demonstrated that the pathways that regulate autophagy under nutrient-scarce conditions are conserved in *Drosophila*. Furthermore, equivalent ATG genes contributing to autophagosome generation in the *Drosophila* fat body model implies that autophagy in this fruit fly species is dependent on preserved features of ATG machinery.

Previous research demonstrated TOR-mediated autophagy across *Drosophila* and mammals. In their study, Kim et al. [205] discovered that Rag GTPases activate TOR when amino acid signaling is present. Experiments on cell cultures from fruit flies and mammals revealed that a reduction in Rag gene expression weakens the impact of amino acids on the TOR pathway. To further verify the role of Rag in the TOR-mediated regulation of autophagy and cell size regulation, they conducted an in vivo testing in the fat body of fruit flies. In a subsequent study, Li et al. [206] explored the regulation of TOR activity and autophagy by Rab and Arf family GTPases. They discovered that these GTPases also regulate TOR, though not through direct interaction with it, unlike Rag.

## 2. Future Directions

Several studies utilizing the *Drosophila* model demonstrated that several signaling pathways and treatments play well-established roles in control of aging and cancer. *Drosophila* researchers have access to an extensive set of tools for investigating autophagy. The remarkable conservation of the autophagy machinery between this fruit fly species and humans has made cross-species research particularly valuable, making possible the discovery of up-to-now uncharted territories in the autophagy pathway. Of particular note, though, it is crucial to use multiple, complementary assays to accurately determine the status of autophagy in *Drosophila*, just as with various other species. Given that this testing model’s cell biology and physiology exhibit striking similarities to those of humans and that there are well-established models for analyzing various pathologies, such as the progression of cancer and neurodegeneration, it has proven to be the ideal system for studying the fundamental mechanisms and regulation of autophagy. With the establishment of cultured cell models for autophagic cell death, bacterial autophagy, and viral autophagy, the amount of research involving RNAi screens for such phenomena should increase in the coming years. Based on recent knowledge gained from autophagy research, this review article will hopefully help us gain a better understanding of autophagy machinery at the molecular level in *Drosophila* and humans. There are, however, still many aspects of autophagy in flies that need to be explored, such as selective organelle degradation and selective autophagy, or xenophagy. Further advancements in the understanding of the function and regulatory pathways of autophagy in *Drosophila* should yield new insights for grasping the significance of this process.

## Figures and Tables

**Table 1:** Genes that are conserved in autophagosome induction, nucleation, and expansion27.

Autophagy Process	Model Organisms/Gene			References
	*Homo sapiens*	*D. melanogaster*	*S. cerevisiae*	
Induction	ULK1, ULK2	Atg1	ATG1	[28,29,30,31,32,33]
	mTOR	dTOR	TOR	[29,34,35,36,37]
	HARBll	Atg13	ATG13	[38,39,40]
	-	-	ATG17	[41,42]
	-	-	ATG29	[43]
	-	-	ATG31	[44]
	FIB200, RB1CC1	CG1347	-	[45]
	ATG101	CG7053	-	[45,46]
Nucleation	BECN1	Atg6	ATG6	[29,47,48,49,50,51]
	PIK3C3	Pi3K59F	VPS34	[47,49,52]
	PIK3R4	Ird1	VPS15	[49,53,54]
	ATG14 (barkor)	CG11877	ATG14	[43,49,55,56]
	UVRAG	CG616	-	[49,57]
	SH3GLB1	endoB	-	[58]
	BCL2	buffy	-	[59,60,61]
	AMBRA1	-	-	[62]
Expansion	ATG2A, ATG2B	Atg2	ATG2	[63,64,65]
	ATG3	Atg3	ATG3	[66,67,68]
	ATG4A,B,C,D	Atg4	ATG4	[69,70,71]
	ATG5	Atg5	ATG5	[32,72,73,74,75]
	ATG7	Atg7	ATG7	[68,69,72,73,76]
	ATG9A	Atg9	ATG9	[74,77]
	LC3, GABARAP, GABARAPL2	Atg8a, Atg8b	ATG8	[67,70,78,79,80,81,82,83]
	ATG10	CG12821	ATG10	[66,73]
	ATG12	Atg12	ATG12	[41,66,73]
	ATG16L1, ATG16L2	CG31033	ATG16	[84,85]
	ATG18	Atg18	ATG18	[77,86,86,87,88]

## Data Availability

Not applicable.
